# The New Deal: A Potential Role for Secreted Vesicles in Innate Immunity and Tumor Progression

**DOI:** 10.3389/fimmu.2015.00066

**Published:** 2015-02-24

**Authors:** Alberto Benito-Martin, Angela Di Giannatale, Sophia Ceder, Héctor Peinado

**Affiliations:** ^1^Children’s Cancer and Blood Foundation Laboratories, Department of Pediatrics, Weill Cornell Medical College, New York, NY, USA; ^2^Department of Oncology and Pathology, Karolinska Institutet, Stockholm, Sweden; ^3^Microenvironment and Metastasis Laboratory, Department of Molecular Oncology, Spanish National Cancer Research Centre (CNIO), Madrid, Spain

**Keywords:** innate immunity, exosomes, tumor progression, tumor surveillance

## Abstract

Tumors must evade the immune system to survive and metastasize, although the mechanisms that lead to tumor immunoediting and their evasion of immune surveillance are far from clear. The first line of defense against metastatic invasion is the innate immune system that provides immediate defense through humoral immunity and cell-mediated components, mast cells, neutrophils, macrophages, and other myeloid-derived cells that protect the organism against foreign invaders. Therefore, tumors must employ different strategies to evade such immune responses or to modulate their environment, and they must do so prior metastasizing. Exosomes and other secreted vesicles can be used for cell–cell communication during tumor progression by promoting the horizontal transfer of information. In this review, we will analyze the role of such extracellular vesicles during tumor progression, summarizing the role of secreted vesicles in the crosstalk between the tumor and the innate immune system.

## Introduction

Cancer is a systemic disease that has mostly been studied by focusing on the primary tumor. Hence, there is little information available as to how the tumor microenvironment influences tumor progression and metastasis. To date, whether a tumor influences its microenvironment or conversely, whether an aberrant microenvironment reinforces tumor progression, is a fundamental question in tumor biology. Metastatic cells conquer new organs after a phenotypic evolution that not only allows them to move from the primary tumor but also to evade innate and adaptive immune responses. Innate immunity protects us against pathogens or potential invaders. However, it is not clear how the innate immune system acts during tumor progression and metastasis. Among the most important questions that remain to be resolved are *whether innate immune cells are present during tumor progression and metastasis and if so, which are their roles*?, *Is the microenvironment involved in the failure of current therapies and if this is the case, can we “educate” our immune system to control or impede metastasis*? In this review, we will focus on how cancer cells interact with the innate immune system during metastasis, highlighting the potential role of secreted vesicles in this process.

## Innate Immunity in Metastasis, the First Line of Defense

There are different factors that can promote tumor progression and eventually metastasis, such as unbalanced growth signaling, alterations to the cell cycle, genomic mutations, and immune evasion. Metastasis is a complex event that accounts for more than 90% of cancer-related deaths, and it reflects the evolution of the primary tumor through its interaction with the microenvironment of local and distant organs (1, 2). Tumor cells migrate from primary tissues by degrading the surrounding tissue and extracellular matrix, spreading through the blood or lymphatic vessels before reaching their newfound land (3). *Lyden and colleagues* defined the novel concept of “*premetastatic niche”* in which stromal cells such as bone marrow-derived cells, under the spell of the primary tumor, are actively involved in the formation of a suitable microenvironment for the metastasis to develop at distant sites (4). Therefore, cancer cells not only need to promote their own immortality and invade propitious territories but they also need to evolve along with the microenvironment and to find strategies that enable them to survive from the constant immune surveillance (5).

Tumors use two major strategies to escape from immune surveillance: immunoediting, in which the least immunogenic tumor cell variants are selected; and active suppression of the immune response along with establishing conditions, within the tumor microenvironment, that facilitate tumor outgrowth (6). Innate and adaptive immunity and their responses must be overcome by tumors in order to evade their effects (7). Escaping from innate immunity, the first barrier of defense against microbial molecules may be one of the earliest events in the progression of the local tumor into a metastatic cancer. However, cancer immunoediting involves the use of the immune system’s host-protective events to promote tumor progression and the escape of cancer cells from immune responses, allowing them to develop immune evasive features (8). Indeed, both innate and adaptive immunity appear to contribute to cancer immunoediting (9).

## The Innate Immune Response Controls the Balance between the Host and Tumor Microenvironment

Tumor development and metastasis are influenced by the stroma, by angiogenesis, and by the innate and adaptive immune system. The concept of tumor immune surveillance was first contemplated by Paul Ehrlich in 1909, postulating that the immune system can restrict the spontaneous growth of transformed cells by identifying and eliminating them (10). About 50 years later, Burnet and Thomas proposed that tumor-associated antigens can provoke an effective immunological reaction (11). This initial theory of immune surveillance was controversial, especially given the evidence that nude mice that lack an intact immune system are not more susceptible to tumor development (12). In 2001, it was shown that lymphocytes and IFNγ collaborate to prevent tumor immunoediting, thereby preventing the selection of less immunogenic tumor cells (13). Natural killer (NK) cells are the immune effector cells that are active in the elimination of transformed cells but that also promote the maturation and migration of dendritic cells (DCs) with enhanced antigen presentation to T cells (14). In cancer, NK cell activity and the ability to infiltrate tumors may be impaired, and for example, chronic exposure to NK group 2 D (NKG2D) ligand-expressing tumor cells alters NKG2D function in NK cells, promoting NK cell evasion by tumor cells (15). Although the activation of immune cells could result in the eradication of transformed cells, chronic activation of innate immunity, like chronic inflammation, might promote cancer development (16). Cancer has been compared to *a wound that never heals* (17), based on the observation that the tumor is in a state of chronic inflammation. Indeed, immune cells, such as macrophages and mast cells, release soluble agents like cytokines and chemokines promoting the migration and infiltration of leukocytes that can contribute to tumor growth (18). Thus, better understanding how innate tumor surveillance occurs could guide tumor immune therapy that would potentiate the immune system to act against cancer through immunomodulatory approaches.

## Extracellular Vesicles and Innate Immunity: A First Contact

**Figure 1:**
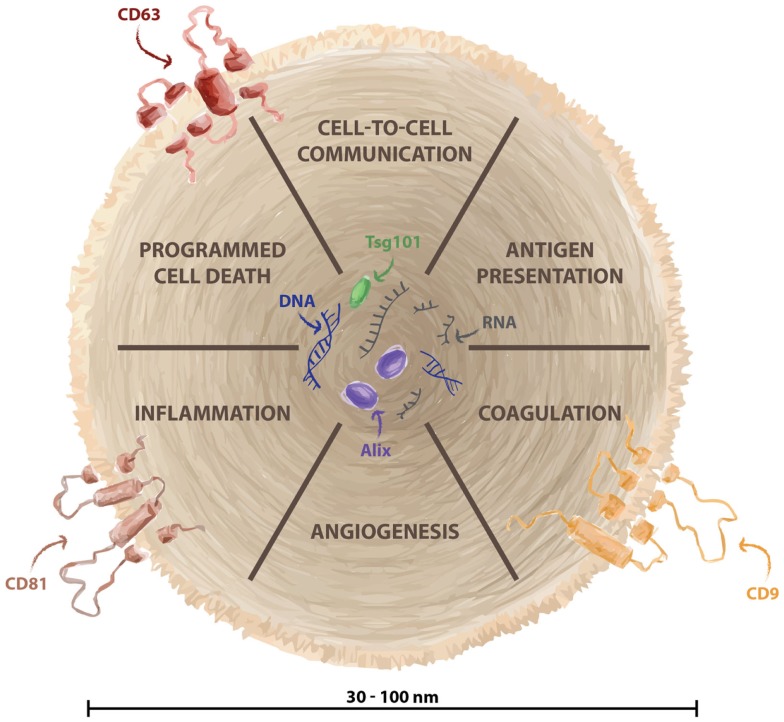
**Biological function of exosomes**. Exosomes are small vesicles (30–100 nm) generated in the endosomal structure and released from several cellular types. They are considered fundamental for cell–cell information exchange, and they contain specific cargo such as DNA, RNA, miRNA, and proteins. Biological functions of exosomes include antigen presentation, regulation of programmed cell death, angiogenesis, inflammation, and coagulation. Exosomes are enriched of specific markers such as CD63, CD81, and CD9.

Most cell types secrete vesicles, and there are many different vesicle types that fulfill a wide range of biological functions. Although the nomenclature is still not fully accepted, one can classify them based on their secretory pathway, and these vesicles can be divided into membrane-derived vesicles and exosomes (19, 20). The term exosome was initially used to name vesicles ranging from 40 to 1000 nm in size that are released by a variety of cultured cells that were defined as “*exfoliated membrane vesicles that may serve a physiological function*” (21). However, this term was later adopted for smaller (30–100 nm) vesicles of endosomal origin that are released as a consequence of the fusion of multivesicular bodies with the plasma membrane, enriched of specific markers such as CD63, CD81, and CD9 (Figure 1) (20, 22). Ectosomes, also called shedding vesicles, microparticles, or microvesicles, are variable in size (0.1–1 μm), origin and cargo. Their secretion occurs through budding from the plasma membrane. Ectosomes are secreted by many cell types including platelets, endothelial cells, and leukocytes, and they are released directly into the blood (23). Extracellular vesicles were thought to discard cellular debris from cells; yet during the last decade, they have been shown to play an active role in cell communication and, in particular, cell–cell communication. They carry molecular determinants of their cell or tissue of origin including DNA, RNA, miRNA, proteins, lipids, and other cargo (Figure 1) (20). The biological functions of exosomes include antigen presentation, regulation of programmed cell death, angiogenesis, inflammation, and coagulation (24). Most studies of exosomes have been carried out in relation to immune or cancer cells (25), as well as studying the potential effects of tumor-derived exosomes in modulating immune interactions. The mechanisms that guide tumor cells to a specific tissue are largely unknown, although there is evidence that tumor cells themselves modulate immune cells and tissues through the secretion of soluble factors and vesicles (2, 26, 27). Exosomes may be vehicles for many different activities exerted over target cells (20). Indeed, we recently showed that melanoma-secreted exosomes transfer information and reprogram bone marrow progenitor cells toward a pro-vasculogenic phenotype in the premetastatic niche, acting through the receptor tyrosine kinase, MET (28). However, the effects of exosome-enriched proteins on immune cells and, in particular, their effects on tumors and innate immunity are not fully understood (29). Although the innate response involves many cell types, it is particularly dependent on basophils and mast cells (inflammation), and on neutrophils and macrophages (phagocytosis). There are evidence of secreted vesicles in most of the innate immunity cellular components. Accordingly, here we shall address the possible involvement of exosomes in modulating the activity of different elements involved in innate immunity.

### Mast cells

**Figure 2:**
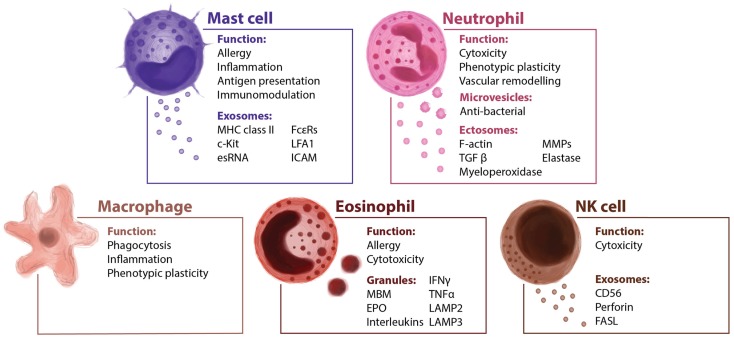
**Innate immune cells and their extracellular vesicles**. The innate immunity is the first barrier of defense against infection and tumoral invasion. It includes humoral factors and many cellular types such as mast cells, neutrophils, macrophages, and NK cells. It is reported that several cellular components of the innate immunity secrete vesicles modulating the direction of the immune response.

**Figure 3:**
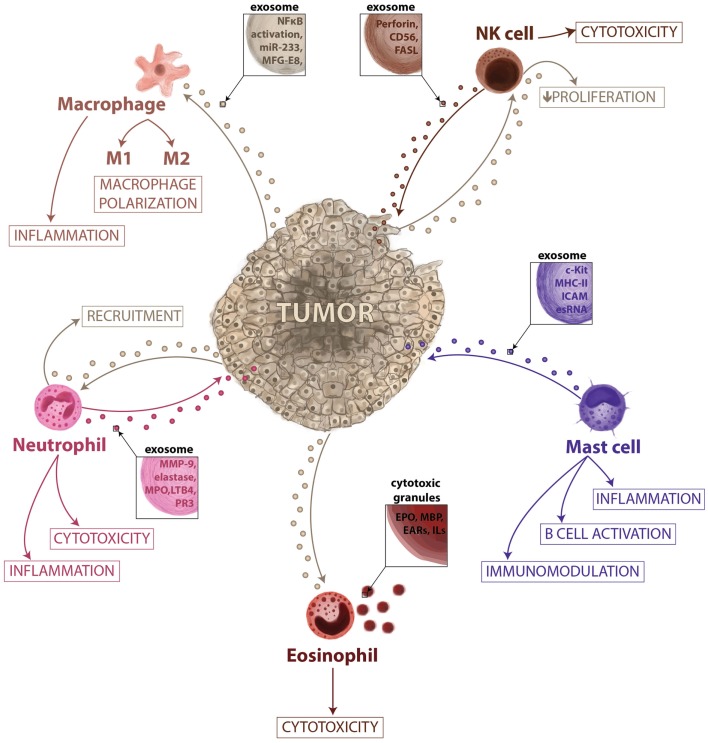
**Vesicle-mediated interactions between tumor and innate immunity**. In this microenvironment–tumor interaction model, tumors secrete exosomes that are responsible for the recruitment of neutrophils and eosinophils, proliferation of NK cells, and activation of macrophages promoting their polarization. When recruited, eosinophils can release their granules having a role in cytotoxic response and immune surveillance. The neutrophils can release vesicles during inflammation activated-process and could have a role in cancer-related inflammation and tissue remodeling. The macrophage-secreted exosomes may regulate the tumor invasiveness and the metastatic behavior; however, specific cargo has not yet been described, while exosomes derived from NK cells may exert a role in immune cytoxicity. Mast cell exosome-derived cargo is described to contain functional mRNAs and small RNAs, immunological proteins and could be involved in immunomodulation, B-cell activation and inflammation.

As well as fulfilling a key role in allergy, mast cells also have other immunomodulatory functions, both pro-inflammatory and anti-inflammatory (30). The pro-inflammatory role of mast cells has been demonstrated to support the tumor microenvironment in different cancer models with different chemokines and cytokines determining the interplay of mast cells with other members of the immune system (31). Stem cell factor (SCF) is a decisive factor in the differentiation, maintenance, and activation of mast cells (32). Indeed, SCF recruits mast cells, and its expression in experimental models of breast and hepatocarcinoma (33). Mast cells are derived from myeloid progenitor cells in the bone marrow, and then undergo differentiation at peripheral sites, where they become specialized into various subtypes (34). Their location allows them to interact with different insults and external threats, so they could be considered as the first line of the immune defense (35). The levels of some proteases present in mast cells are related to tumor progression. Mast cells release proteins that have been correlated with microvessels density, tumor progression, and angiogenesis such as histamine, which could increase new vessels permeability, promoting leakiness (36, 37). Similarly, MMP-2 and MMP-9 stored in mast cell granules, or the secretion of FGF-2, VEGF, TGF-β, or TNF-α could be involved promoting angiogenesis (38). Mast cells have been reported to release exosomes that contain different proteins, providing an additional mechanism of intercellular communication (39–41). The content of these vesicles has been analyzed in detail and the mast cell exosome-derived cargo identified. Mast cell-derived exosomes contain immunologically determinant factors such as MHC class II proteins (42), co-stimulatory (CD86, CD40, CD40L), and adhesion-related (LFA-1, ICAM-1) molecules (40) (Figure 2). They also include CD13, ribosomal protein S6 kinase, annexin V, cdc25, and phospholipases, together with other interacting proteins like aldolase A and heat shock protein 70 (43). The receptor Fcε-RI subunits alpha and beta have been detected in exosomes (44), contributing to the idea that secreted vesicles could amplify immune modulation (Figure 3). Bone marrow-derived mast cells can induce resting B cells to proliferate and to produce IgM (45). What was thought to be a non-contact mechanism was described later as exosome-mediated interaction. These mast cell-derived exosomes are responsible of inducing B-cell production of IL-2, IL-12, INF-γ, IgG1, and IgG2, but not IL-4 (40). Functional mRNAs and small RNAs, including microRNAs have also been found in mast cell-derived exosomes (Figure 2) (41, 46). The RNA contained in the mast cell-derived exosomes was first described by *Lötvall and Valadi* as exosomal shuttle RNA (esRNA), being functional and transferring information between cells (47). The evidence suggest a role for exosomes in transferring relevant information between stromal and tumor cells (28), exhibiting new potential communication mechanisms (Figure 3).

Although the role of mast cells in cancer has been studied widely, this issue is still not yet fully understood. An increase in the mast cell population is associated with poor prognosis in many different cancers, including oral squamous carcinoma, Hodgkin’s lymphoma, diffuse large B-cell lymphoma, and prostate cancer (48–52). A positive correlation was described in prostate cancer, colon, and breast cancer (53). The location of mast cells might be an essential factor in their role in tumor rejection or progression. In non-small cell carcinoma, intra-tumoral detection is associated with improved survival (54). In melanoma, mast cell accumulation in the margins of the tumor has been observed (55). c-Kit and Fcε-R1, phenotypical markers for mast cells, are involved in tumor progression and exosomal communication. Interestingly, c-Kit-containing exosomes have been described as a mechanism for progression in lung adenocarcinoma enhancing tumor cell proliferation (56). These data, together with our recently published data about the influence of c-MET secreted on tumor-derived exosomes to bone marrow progenitor cells during melanoma metastatic progression (28), suggest that horizontal transfer of oncoproteins between tumor and bone marrow-derived cells by exosomes play central role in tumor progression. However, the actions of these exosomal proteins in the environment and their role in establishing either a premetastatic niche or promoting tumor growth are yet to be revealed. Secretion of exosomes is a novel mechanism that expands their role far beyond their already known role in allergy. Increasing body of evidence suggest that mast cell-derived exosomes as potential “modulators” of the crosstalk between immune cells and stromal cells. Mast cell-secreted exosomes may have a double-edged function, while they can act through stimulating the immune system during the early events of cancer or when in contact with foreign substances, they could also be involved in the tumorigenic progression by the recruitment of macrophages, fibroblasts, and blood vessels that contribute to tumor growth by secreting a milieu of molecules.

### Neutrophils

Neutrophils are key mediators of the innate immune system, and their activation is essential to protect the host system against infections and to promote healing (57). The short life span of neutrophils and their specific differentiated phenotype has masked their role in cancer-related inflammation, which is why they have been largely ignored. Similar to macrophages, the so-called tumor-associated neutrophils (TAN) can exert pro-tumoral as well as anti-tumoral effects. Evidence from animal models suggests that neutrophils can be polarized toward distinct phenotypes in response to different tumor-derived signals (58). The role of neutrophils in tumor progression is not fully understood. They gather in metastatic carcinomas modifying the tumor microenvironment by secreting factors like G-SCF or MMP-9 among others (59, 60), and promoting therefore angiogenesis and vascular remodeling. Tumor exosomes act promoting tumor growth through bone marrow progenitor cells education and through neutrophils recruitment (61). Macrophages and DCs are capable of secreting LTB4-producing exosomes (62), which may induce neutrophils recruitment. This calling effect is performed in a gradual fashion that might act like an exosomal gradient (63). Cytokines and secreted factors stored in neutrophils granules may play a role in tumor progression (Figure 3). Epithelial lung cancer cells uptake neutrophil elastase (NE) and favor tumor cell proliferation through the hydrolysis of IRS-1 (64). The acquisition of NE by cancer cells confers them a previously unknown mechanism of anti-tumor adaptive immunity (65). Fridlender et al. have demonstrated that TGF-β drives neutrophils to acquire a pro-tumorigenic N2-phenotype, whereas its inhibition enhances the emergence of an anti-tumorigenic N1-phenotype, characterized by cytotoxic activity on cancer cells and an immunostimulatory profile (i.e., high levels of TNF-α, CCL3, and ICAM-1) (58). Human neutrophils release microvesicles with antibacterial properties, and their cargo, in terms of antimicrobial proteins (Figure 2), is different in the depending on the stimuli (66). When activated *in vitro* and *in vivo*, in local and systemic inflammation, neutrophil microvesicles are shed through an exocytotic process and they have been denominated ectosomes (Figure 2) (67). These vesicles contain cytosolic F-actin indicating their outside-out orientation, but their content and unique characteristics suggest a role in inflammation (68). Ectosomes activate multiple signaling pathways in neutrophils and macrophages, leading to TGFβ1 secretion, and may play a role in macrophage and TAN polarization (69). Neutrophil-derived ectosomes contain TGF-β, MMP-9, myeloperoxidase, proteinase 3, or elastase (Figures 2 and 3), postulating them as candidates for determinant roles in inflammation and cancer signaling (Figure 3) (70). While the classical role of neutrophils and their secreted vesicles has been described as antibacterial agents, recent data suggest that they could also play a role in inflammation and influence tumor immunosurveillance. Secretion of molecules such as proteases and elastases would impact in the ECM remodeling necessary for tumor progression, recruitment of neutrophils, and release of their exosome cargo to the tumor microenvironment could therefore be involved not only in primary tumor progression but also in the formation of metastatic foci.

### Macrophages

Macrophages are closely involved in the inflammatory responses observed during cancer and metastasis (Figure 2). Many observations indicate that tumor-associated macrophages (TAM) have several pro-tumoral functions, including the expression of growth factors and MMPs, the promotion of angiogenesis, and the suppression of adaptive immunity (16). Tumor-secreted exosomes promote the activation of macrophages, as evident by NF-κB activity (71). Cancer cells secrete factors that modulate macrophage activation and polarizations into M2 macrophages (Figure 3). However, the mechanisms that mediate such polarizations are not clear. Glycoprotein MFG-E8 is associated with the suppression of pro-inflammatory responses, and it is more concentrated in exosomes from the tissue and serum of prostate cancer patients (72). Indeed, the administration of an antibody against MFG-E8 significantly attenuates M2 polarization (72). Therefore, one of the potential roles of tumor-derived exosomes, in this context, would be the regulation of M1/M2 polarization. The contribution of exosomal miR-233 in autocrine differentiation of macrophages has also been proposed (Figure 3) (73). Macrophages infected with intracellular pathogens such as *Mycobacterium tuberculosis*, *Mycobacterium bovis BCG*, *Salmonella typhimurium*, or *Toxoplasma gondii* release exosomes that contain pathogen-associated molecular patterns (PAMPs). These exosomes have immunomodulatory properties over naïve macrophages and neutrophils both *in vitro* and *in vivo* (74). Amoeboid prostate cancer cells have also been described to secrete miR-125a, which suppresses AKT1 expression and hence, the proliferation in recipient human peripheral blood mononuclear cells and macrophages (75). It was recently described that breast cancer-derived exosomes are capable of inducing an inflammatory response in macrophages through NF-κB activation, as seen in other pathologies (76), and that this may contribute to metastasis (77). Tumors may use exosome-mediated macrophage polarization to reinforce the escape of tumors from immune surveillance and in this sense, macrophage-derived exosomes regulate integrin β1 expression in endothelial cells *in vitro*, eventually promoting endothelial migration (78). Its contribution to vascular leakiness and tumor invasion is plausible but not determined yet. Macrophage-secreted exosomes may regulate the invasiveness of breast cancer cells through the delivery of oncogenic miRNAs. For example, miR-223 was detected within the exosomes promoting the invasion of breast cancer cells via the Mef2c-β-catenin pathway (73). These data suggest that exosome communication and transfer of information between tumor cells and macrophages could be actively involved in regulating tumor progression (Figure 3) (79). Although macrophage-secreted exososomes could play an active role regulating phagocytic events and degradation of foreign substances, there are a reduced number of evidence of their physiological function. However, their role in cancer is better defined, the continuous stimulation by the primary tumor normally overcome the macrophage-driven immunosurveillance. One potential consequence of the massive secretion of exosomes by macrophages in pathological scenarios would be the generation of an inflammatory microenvironment, which may ultimately result in an enhanced metastatic burden.

### Eosinophils

Eosinophils are traditionally considered to be effector cells in allergic diseases and infections due to their cytotoxic potential (Figure 2). They are involved in the inflammatory response due to the production and release of a wide set of cytokines and mediators (80). Their granules contain highly cytotoxic proteins, such as major basic protein (MBP) or eosinophil peroxidase (EPO) (Figure 2), and they can present antigens, release other inflammatory mediators and participate in the regulation of the adaptive immune response. Like members of the innate immune system, eosinophils have been observed in the tumor-infiltrating area, and their presence is correlated with good prognosis, irrespective of other prognostic factors such as stage, age, and histological grading (81). It is not fully understood what mechanisms drive eosinophil recruitment to the tumor site (82), although some tumor cells secrete IL-5 and IL-3 that modulate the differentiation and maturation of eosinophils and mast cells (83). A variety of studies indicate that eosinophils might be more than just an effector of tumor surveillance, indicating that they are capable of processing insults and of orchestrating a quick and selective response, mediated through the release of their contents (84).

Eosinophilic granules have been described intensely, and some of their features are similar to those features of exosomes (Figure 2) (85). Specific granules of human eosinophils are notable for their storage role for various proteins, such as EPO, MBP, and eosinophil-derived neurotoxin (Figure 3), all of them with potent cationic properties. In addition, these granules contain cytokines, chemokines, and growth factors, including IL-2, -3, -4, -5, -6, -10, -12, and -13, IFN-γ, TNF-α, NGF, GM-CSF, SCF, TGF-α, RANTES (CCL5), eotaxin-1 (CCL11), and CXCL5 (86), many of which are present in other immune secreted vesicles. In contrast to exosomes, eosinophil granules are larger in size (500–1000 nm) and express lysosome-associated membrane glycoproteins such as LAMP-2 (CD107b) and LAMP-3 but vesicle-associated membrane protein-2 is not expressed on human eosinophil granules (Figure 2) (87). Despite the selective and well-controlled secretion of cytokines and cationic proteins, there is no evidence that cell-free eosinophilic granules can function as antigen-presenting structures or express MHC class II molecules. Little is known at present about eosinophil-derived exosomes or secreted vesicles, and even less regarding their immunomodulatory functions. The pro-inflammatory and cytotoxic properties of eosinophils make them an interesting target for cancer immune surveillance. Indeed, if cancer cells could inactivate eosinophils or take advantage of other leukocytes recruitment that eosinophils promote, they might have a means to elude one of the main effector cells of the innate immune system. While the role of eosinophil-secreted exosomes is yet to be defined, they may have an important role in the pathogenesis of several diseased such as asthma and tumor progression. Their physiological role executing cytotoxic effects could be likely modulated by pro-inflammatory factors. Moreover, the release of their cargo together with mast cells could be considered as a future biomarker of chronic pathologies characterized by an inflammatory microenvironment.

### NK cells

Natural killer cells are among the most powerful effectors lymphocytes in the immune system: 15% of all circulating lymphocytes are NKs, and they are also found in many peripheral tissues. Different factors recruit NK cells to the peripheral tissues, such as IL-12, IL-15, IL-2, IFN-α, or β, and their role in innate immunity and in anti-tumor defense has been studied (88). Recent evidence shows that NK cells isolated from the blood of healthy donors release exosomes expressing proteins such as CD56 and that they contain the killer protein perforin (Figure 2) (89). Exosomes released by NK cells may exert cytotoxic activity against tumor target cells, pointing to an important role of NK cell-derived exosomes in immune surveillance (Figure 3) (89). NK-derived exosomes contain FasL, a traditional pro-apoptotic ligand (90) that has been implied in tumor tolerance (91), and perforin, a granzyme implicated in tumor and immune homeostasis (92), which lead to literary name NK-derived exosomes as “nanobullets” (93). The proliferation and cytotoxic activities of NK cells is impaired by tumor-derived exosomes, both *ex vivo* and *in vitro*, and this effect has been correlated to the growth of the tumor (94). In fact, exosomes released by human pancreas and colon carcinoma cells in culture express high levels of Hsp70, and they can stimulate NK cell activity, inducing their migration and cytolytic activity (95). Other reports described that tumor-derived exosomes can also promote tumor immune evasion by impairing NK cells effector functions in human breast cancer, mesothelioma, and various types of blood cancers (96). NKG2D is one of the most potent activating receptors expressed on the surface of NK cells, and it plays an important role in activating the anti-cancer immune response through an interaction with stress-inducible NKG2D ligands (NKG2DL) on transformed cells (97). However, cancer cells have developed numerous mechanisms to evade the immune system, NKG2D ligand-bearing exosomes downregulate NKG2D receptor-mediated cytotoxicity *in vitro* (98). Current tumor immunotherapies may produce severe side effects, although a Cbl-b-knockout mouse and mice treated with a TAM blocker do not exert severe signs of inflammation or autoimmunity, representing a therapeutic window for Cbl-b/TAM receptor inhibition to mediate tumor rejection without provoking serious cytotoxicity (99). The most recent data in this field suggest that NK cell-derived exosomes play an active role in the regulation of immune surveillance and homeostasis. Moreover, due to their cytotoxic activity, NK-derived exosomes are probably the best example so far of “nanobullets” that could possibly be used in future therapeutic approaches against different pathologies, including tumors.

## Reinterpreting Cancer, Lessons Learned from Parasite World

When parasites infect an organism, they infiltrate its tissues and organs, trying to avoid the host’s defense systems. Eventually, they will find their niche, proliferate, and develop a full infection if the immune response is insufficient. This behavior resembles the colonization of metastatic sites by cancer cells and their adaptation to the metastatic niche, suggesting that both processes may share similar mechanisms (26). Both microorganisms and tumor cells need to interact with the innate immune system and evade leukocyte surveillance in order to achieve successful colonization. A comprehensive study of the tapeworm genome identified evolutionary adaptations that may influence such colonization, affecting metabolism, detoxification mechanisms, loss of homeobox genes, empowered stemness, and altered cadherins (100). As such, some enzymes involved in these adaptations may be relevant targets for anti-parasitic drug development and could also possibly be used as anti-cancer therapies. For example, anti-parasitic agents such as mebendazalone have been described as a treatment of chemotherapy-resistant malignant melanoma (101) and likewise, different classes of anti-malarial drugs (artemisinins, synthetic peroxides, and DHFR [dihydrofolate reductase] inhibitors) have strong anti-proliferative activity on cancer cells (102). These observations have encouraged further research into the possible anti-cancer applications of new small compounds with anti-parasitic activity. Importantly, there are cases in which parasitic infections are the cause of cancer, expanding our perception of the relationship between parasites and cancer (103, 104). While such a relationship of carcinogenesis is not fully understood, it may be related to aberrant innate immunity and chronic inflammation.

Parasites secrete exosomes that interact with the innate and adaptive immune systems (105). Exosomes from *Leishmania donovani* modulate the monocyte response to IFN-γ, promoting IL-10 production and inhibiting TNF-α. *L. donovani* is the parasite responsible for the most severe leishmaniasis, and it infects the mononuclear phagocyte system, affecting macrophages in the spleen, liver, and bone marrow via a Rac1- and Arf6-dependent process (106). *M. tuberculosis* is another microbe that uses a strategy that cancer cells may mimic. Virulent *M. tuberculosis* evades innate immunity by inhibiting apoptosis and triggering necrosis of host macrophages, and escaping from adaptive immunity by delaying its initiation (107). This interaction with macrophages might me mimicked by cancer cells. Epigenetic modulation of the immune response has also been described in *Plasmodium falciparum*, the protozoan parasite responsible for the human malaria. *P. falciparum* has 60 *var* genes encoding distinct antigenic forms of the virulence protein PfEMP1 (*P. falciparum* erythrocyte membrane protein 1). The parasite expresses only one *var* gene at any time point during infection to avoid detection by the immune system, yet the mechanism controlling the silencing of the other 59 *var* genes is unknown (108). An epigenetic mechanism is thought to be involved in this silencing, whereby the histone lysine methyltransferase PfSETvs controls, which gene will be expressed and thus, enables the parasite to evade the immune system (108). Therefore, the transfer of epigenetic modulators through exosomes may participate in the avoidance of immune surveillance.

## The Role of Innate Immunity in Specific Pathologies

Activation of the innate immune system, or the inhibition of pro-tumorigenic and inflammatory cells, can produce anti-tumoral effects in immunosuppressed cancer patients. Here, we discuss specific cancer types that provide evidence of the crucial role mast cells, macrophages, and NK cells could play in tumor formation and progression, as well as the targeted immunotherapy currently available in such cases.

### The role of mast cells and macrophages in neurofibromatosis-1 related tumors

Although important roles of the tumor microenvironment and mast cells in the initiation and progression of neurofibroma have been described (109), their interactions remain poorly understood. The hypothesis that in neurofibroma, tumorigenic cells do not grow in isolation but rather the microenvironment contributes critically to their formation, suggests that exosomes may possibly mediate this process. So far, there is a lack of information of the role of secreted vesicles in the communication between tumor and stromal cells in neurofibroma progression, a complication of neurofibromatosis-1 (NF1) (110). Neurofibromas are formed in association with peripheral nerves and as well as containing large collagen deposits, they are composed of Schwann cells, fibroblasts, vascular cells, and mast cells. Tumor progression requires complex interactions between Schwann cells and NF1 heterozygous (NF1^+/−^) cell lineages in the tumor microenvironment including innate immune system cells (111). Exposure of NF1^+/−^ mast cells to conditioned media from Schwann cells and mast cells *in vitro* provoked hypersensitivity (112, 113), suggesting that activated mast cells can release inflammatory cytokines, growth factors, and other components such as extracellular vesicles, which may stimulate tumorigenic Schwann cell transformation and support the recruitment of macrophages, fibroblasts, and blood vessels. In addition, mast cells can secrete VEGF, a potent stimulant for Schwann cell proliferation and survival. VEGF inhibits the differentiation and functional maturation of DCs by suppressing NF-κB in hematopoietic stem cells, and by downregulating the anti-tumor response (114). VEGF has been demonstrated to be carried in tumor-shed vescicles and to be released in a bioactive form (115). Therefore, mast cell-derived exosomes are good candidates to mediate tumoral transformation. Several studies have tested the hypothesis that NF1 heterozygotes enhance angiogenesis and may promote neurofibroma formation (116, 117); nevertheless, none of these studies showed so far the role of secreted vesicles in the communication between different cell types. We postulate that tumor-secreted exosomes could play a novel role in cell–cell communication with mast and endothelial cells during neurofibroma progression.

Currently, many cancer therapies targeting innate immunity have been studied in the context of NF1 and some such trials are still ongoing (Table 1). Some of these drugs may target not only tumor molecules or soluble factors but also tumor-shed vesicles. NF1 patients with symptomatic plexiform neurofibromas had decreased tumor volumes in a phase 2 study after treatment with kinase inhibitor imatinib mesylate, which targets the c-Kit receptor, ABL, BCR-ABL, and PDGFRα (118). Based on the hypothesis that mast cell secretions contribute to the growth and associated symptoms of neurofibromas, ketotifen (a mast cell granule stabilizer) was shown to reduce neurofibroma-associated symptoms and tumor growth. A reduction in exosomes release might be associated to the treatment with granule stabilizers. A subsequent multiphase trial confirmed the symptomatic control with this agent, yet without neurofibroma reduction (119). As malignancy develops, macrophages are recruited at higher densities to peripheral nerves and neurofibromas in mice and human beings when NF1 is inactivated in Schwann cells (120). PLX3397 (Plexxikon) is a novel small molecule that selectively inhibits CSF1R, c-Kit, and mutant FLT3 kinases, by targeting key components of both the tumor and its microenvironment (such as macrophages, osteoclasts, and mast cells). The effects of PLX3397 in neurofibroma formation indicated that macrophage infiltration seems to have both anti- and pro-tumorigenic roles depending on the disease stage (120). Currently, the lack of efficient treatment options for neurofibroma patients is due to the lack of understanding the mechanisms how it progresses. Defining if exosomes play a role in neurofibroma-MPNST transformation will help in identifying individuals who may be at a high risk of progression. Early diagnosis of malignant transformation is key to design therapeutic approaches in NF1 patients, and exosomes could be the missing biomarker for neurofibromatosis.

**Table 1:** **Current clinical trials in cancer involving innate immunity modulation**.

Agents	Molecular targets	Target cells (innate immunity)	Tumor	Phase	Reference
Imatinib	c-KIT, ABL, BCR-ABL, PDGFRA	Mast cells	PN NF1	2	(118)
Ketotifen fumarate	Blocking of histamine binding (H1 receptor), inhibition calcium-dependent vesicle degranulation of activated mast cell	Mast cells	Neurofibromas NF1	Controlled multiphase trial	(119)
PLX3397	CSF1R, KIT, FLT3-ITD	Machrophages Mast cells	PN NF1 (Nf1flox/flox mouse model)	P (*in vivo*)	(120)
L-MTP-PE, Mifamurtide Mepact	NOD2, NLRP3 (NF-kB, MAPKs activation)	Macrophages	Osteosarcoma	3	(121, 122)
Haploidentical NK cells + IL-2 + CT	Cytolytic activity	NK	Neuroblastoma	1	clinicaltrials.gov (NCT00698009)
Allogeneic NK cells + 3F8 + CT	Cytolytic activity	NK	Neuroblastoma	1	clinicaltrials.gov (NCT00877110)
Humanized Anti-GD2 (hu14.18K322A) ± NK cells + CT	Cytolytic activity	NK	Neuroblastoma	1	clinicaltrials.gov (NCT01576692)
Humanized anti-GD2 (Hu3F8) + GM-CSF	Disialoganglioside GD2 + GM-CSFR	NK cells, monocytes/macrophages	Neuroblastoma	1	clinicaltrials.gov (NCT01757626)
Rhu-GM-CSF + CT	GM-CSFR	Monocytes/macrophages, CD4-T,NK, DCs	Colorectal cancer	2	(123)
Dendritic cells + PANVAC or PANVAC + GM-CSF	Carcinoembryonic antigen and mucin-1	Tumor antigen specific T cells	Colorectal cancer	2	(124)

*PN, plexiform neurofibroma; P, preclinical; MPNST, malignant peripheral nerve sheath tumor; PVNS, pigmented villonodular synovitis; GBM, glioblastoma; TMZ, temozolomide; RT, radiotherapy; CT, chemotherapy*.

### The role of tumor-infiltrating macrophages in osteosarcoma

Osteosarcoma is the most common primary malignant tumor arising in the bone, and it occurs most frequently in adolescents. In patients with osteosarcoma, the lungs are the most common site of distant metastasis (125) and the most common site for recurrence.

The immune system is likely to play an important role in osteosarcoma progression, and indeed, the presence of infiltrating macrophages is associated with improved survival and decreased incidence of metastasis. However, the role of vesicles in the osteosarcoma/immune system interaction has not been demonstrated yet. Thus, strategies that target innate immunity seem to represent a promising approach to treat this tumor (Table 1). Mifamurtide (liposomal muramyl tripeptide phosphatidyl ethanolamine: L-MTP-PE, Mepact), is a modulator of innate immunity that stimulates the anti-tumoral effect of monocytes and macrophages (121). The results of an US randomized phase III trial (INT 0133) of L-MTP-PE associated to chemotherapy to treat osteosarcoma reported a significant interaction of the combination L-MTP-PE/ifosfamide (122). M1 and M2 macrophages appear to inhibit growth of osteosarcoma cells *in vitro* and, in particular, the M1 subpopulation is activated by L-MTP-PE when associated with IFNγ and the M2 macrophages with IL-10 in the presence of anti-EGFR cetuximab involved in antibody-dependent phagocytosis (126). Osteosarcoma cell secrete exosomes, and they might interact with macrophages and other immune cells, as has been described above. We postulate a role of exosomes as mediators of immune response in osteosarcoma, and other studies will be needed to develop new drugs that enhance the innate immune system in these patients.

### NK cells in neuroblastoma treatment and progression

Natural killer cells recognize and eliminate transformed cells that downregulate the expression of human leukocyte antigen (HLA) class I molecules (127). The efficiency of NKG2D in NK cell-mediated cytotoxicity is strictly correlated with the expression and surface density of the MHC class I-related chain (MICA or NKG2D ligand) on target tumor cell (128). Neuroblastoma is a neuroendocrine tumor, arising from neural crest element of the sympathetic nervous system and represents the most common extracranial solid cancer in childhood. Patients with neuroblastoma show high serum soluble MICA (sMICA)associated with the downregulation of surface NKG2D in normal peripheral blood CD8+ cells, decreased NK-mediated killing of MICA+ neuroblastoma cells, HLA class I antigen-deficiency and defects in antigen processing (129). Furthermore, neuroblastoma may evade the immune system by downregulating activating ligands for the immunoreceptor NKG2D expressed by cytotoxic T lymphocytes and NK cells (130). It is reported that prostate carcinoma cell line secrete MICA in association with exosomes, and this may contribute to immune escape mechanism of different tumoral cells (131). Tumor-derived exosomes can both stimulate NK cell activity or promote tumor immune evasion by impairing NK cells effector (132). Neuroblastoma cell lines are known to release exosomes (133), and their role in immune surveillance should be investigated, particularly as they might represent useful targets for immunotherapeutic approaches (134). NK-derived exosomes increase NK cytotoxic potential over the tumor. NK cells have been successfully used in adoptive immune cell infusions to cure various advanced or metastatic tumors inducing a graft-versus-tumor (GVT) effect (135), so NK-derived exosomes could be used as drugs to reduce neuroblastoma tumor size. At the moment, different trials are ongoing to evaluate the efficacy of NK cell infusion in patients with relapsed or refractory neuroblastoma, in conjunction with the use of anti-GD2 antibodies (Table 1, clinicaltrials.gov).

### Innate immunity and colorectal cancer

Colorectal cancer (CRC) is the third most commonly diagnosed cancer in males and the second in females. The risk of developing CRC is influenced by both environmental and genetic factors and patients with inflammatory bowel diseases (IBDs), among which the most common forms are Crohn’s disease and ulcerative colitis, present a higher risk of developing colitis-associated CRC (136). We postulate that the continuous secretion of exosomes and recruitment of stromal cells promoted by the constant inflammation in IBD patients may be a risk factor in the development of CRC. However, the majority of CRCs develops without any apparent pre-existing inflammatory pathology. Commensal microorganisms contribute to host defenses, controlling intestinal inflammation, and maintaining intestinal homeostasis through crosstalk with the innate immune system. The benefits of targeting innate immunity in colon cancer has been studied, although a phase 2 study of recombinant human GM-CSF administrated pre-operatively to colon cancer patients failed to find an association with beneficial immune function (123). Another trial compared the effectiveness of two different vaccines (DCs modified with PANVAC or PANVAC plus GM-CSF) in the treatment of patients with CRC (124) showed that DCs and poxvector vaccines have similar activity but that the survival of vaccinated patients was longer than for unvaccinated patients. Patients with CRC present higher serum exosomal levels if compared with controls, but their role in the innate immunity is not yet described (137). Exosomes (or tolerosomes) have been shown to contribute to maintaining tolerance to food antigens in the gastrointestinal tract (138). Therefore, exosomes released by immune cells not only play an immunostimulatory effect but they are also involved in the maintenance of immunological tolerance, which may be lost in inflammatory diseases, thereby contributing to cancer development. Furthermore, a study showed significantly higher levels of exosomal miRNAs in the serum of patients with colon cancer than in healthy controls, levels that were substantially downregulated after surgical resection of the tumors (137). Inflammation and cancer progression are very closely related in CRC progression, and exosomes are key players in this interplay.

## Conclusion

The immune system performs tumor immune surveillance to inhibit tumorigenesis and prevent the establishment of a premetastatic niche. In this context, it remains unclear which are the effector molecules that allow cancer cells to evade immune surveillance and that drive immunoediting and tumor promoting inflammation through interactions in the tumor microenvironment. In this sense, it would be of interest to determine *how immune activity can be harnessed for clinical benefit*. There is new evidence focusing on the interaction between the innate immune system and effector molecules secreted by tumor cells. However, to date, there is limited data about as to how tumor-secreted vesicles can act as “first messengers” to prepare the metastatic niche and how innate immunity reacts to that threat. One critical point in this field is that the majority of the studies up to date involves normally *ex vivo* manipulations on extracellular vesicles that do not necessarily reflect the physiological situation. Similarly, in most of the studies, it is hard to determine the biological role of extracellular vesicles *in vivo a*nd difficult to interpret. Furthermore, very often the exact source of extracellular vesicles is not well known, and the assays performed made the mechanistic analysis difficult to interpret. There is scarce information yet regarding the secreted vesicles actions in physiological conditions although progression rates in the field are promising. A better characterization of the source and more mechanistic assays would be required to define the specific role of extracellular vesicles secreted by each cell type. *Do tumor-secreted vesicles regulate the behavior of the innate immune system during metastasis*? *Can they act as an invisible threat to orchestrate metastatic niche formation*? *Can they be stopped*? Further research is needed to define the role of secreted vesicles in metastasis and how this information “educates” the tumor microenvironment.

## Conflict of Interest Statement

The authors declare that the research was conducted in the absence of any commercial or financial relationships that could be construed as a potential conflict of interest.
